# Genomic Medicine: Lessons Learned From Monogenic and Complex Bone Disorders

**DOI:** 10.3389/fendo.2020.556610

**Published:** 2020-10-09

**Authors:** Katerina Trajanoska, Fernando Rivadeneira

**Affiliations:** Department of Internal Medicine, Erasmus MC University Medical Center, Rotterdam, Netherlands

**Keywords:** genomic medicine, osteoporosis, complex diseases, monogenic bone disorders, drug repurposing, Mendelian randomization

## Abstract

Current genetic studies of monogenic and complex bone diseases have broadened our understanding of disease pathophysiology, highlighting the need for medical interventions and treatments tailored to the characteristics of patients. As genomic research progresses, novel insights into the molecular mechanisms are starting to provide support to clinical decision-making; now offering ample opportunities for disease screening, diagnosis, prognosis and treatment. Drug targets holding mechanisms with genetic support are more likely to be successful. Therefore, implementing genetic information to the drug development process and a molecular redefinition of skeletal disease can help overcoming current shortcomings in pharmaceutical research, including failed attempts and appalling costs. This review summarizes the achievements of genetic studies in the bone field and their application to clinical care, illustrating the imminent advent of the genomic medicine era.

## Introduction

The concept of “precision” or “personalized” medicine, i.e., individualized prevention and treatment tailored to a patient's individual needs is not new. What is clear is that it has gained notorious popularity over the past decade, fuelled by the advances in human genomics, setting the ground for the field of “Genomic Medicine.” The biggest breakthroughs in the field were achieved with the emergence of the genome-wide screens across large study populations which have uncovered many novel molecular mechanisms underlying rare and common human diseases. This review summarizes the achievements of genetic studies in the bone field (both for monogenic and complex traits) and their application to clinical care, illustrating the imminent advent of the genomic medicine era.

The translation of genomic findings to the clinic has been particularly successful for the diagnostic screening and treatment of rare monogenic diseases. For instance, *ivacaftor* is a drug widely used in clinical practice to treat patients with cystic fibrosis; the compound targets directly the gene defect in patients with at least one *G551D* mutation in the *CFTR* gene (1). Gene therapy has also been introduced for several other monogenic diseases such as inherited immune deficiencies (2), hereditary blindness (3), hemophilia (4), and beta-thalassemia (5) among others. Similarly, tremendous progress has been made in cancer research with the development of the chimeric antigen receptor T cell (CAR-T) immunotherapy which acts as a “living drug” against cancer cells (6). In contrast, the translation of genomic discoveries for common diseases has lagged behind due to their complex nature, i.e., involvement of numerous genetic variants of weak effects and large environmental influence.

## What Have We Learned From Monogenic Skeletal Disorders?

In general, monogenic diseases arise due to mutation in a specific gene and are responsible for sometimes life-threatening abnormalities across different bodily systems, including the musculoskeletal. Based on the inheritance pattern, monogenic diseases can be classified as recessive or dominant. These diseases have been extensively described in previous reviews (7–10). Below we will focus mainly on those musculoskeletal conditions where novel treatments have been developed.

### Osteogenesis Imperfecta

Osteogenesis imperfecta (OI) is a group of genetic disorders characterized by weak bones, of which most are of autosomal dominant inheritance (types I, II, III, IV, V, and VI); while a few are inherited in an autosomal recessive manner (types VII, VIII and some cases of type III). Most of the mutations are present in either the *COL1A1* or *COL1A2* genes, which encode proα1 (I) and proα2 (I) collagen chains (11). Mutations in these genes affect the composition of the bone matrix and bone architecture, due to alterations in the collagen cross-links and of collagen 1 triple helix formation. These material properties of collagen 1 fibrils constitute strong determinants of bone strength, and are affected to varying degrees in OI (type I-IV). Notably, the *COL1A1* gene has also been associated with variation in bone mineral density (BMD) in the general population (12).

Patients with OI display wide range of skeletal features such as low bone mass, increased bone fragility and fracture risk, physical disability, and chronic pain which altogether decrease the quality of life. OI treatment is based on disease severity and patient's specific symptoms. While still pending to reach clinical application, several efforts have been made to correct the underlying genetic defect of OI. Chamberlain et al. using adeno-associated virus (AAV) targeting vector have successfully inactivated mutant *COL1A1* (13) and *COL1A2* (14) genes in OI mesenchymal stem cells (MSCs), thus restoring the production of normal type 1 procollagen. Induced pluripotent stem cells (iPSCs) generated from gene-targeted MSCs isolated from OI patients were able to differentiate to osteoblasts and produce normal collagen as well (15). Recently, using CRISPR-Cas9 several iPSC lines have been created to explore the OI mechanisms and therapeutic approaches *in vivo* (16, 17). Furthermore, MSC transplantation in OI patients has showed promising results leading to increased BMD and decreased fracture risk after transplantation in both mice and humans (18–21). In 2016, EU approved the Boost Brittle Bones Before Birth (BOOSTB4) study whose sole aim is to perform Phase I/II clinical trials on the safety and efficacy of prenatal and/or postnatal transplantation of fetal-derived MSC for severe forms of OI (Type III and IV) (22). Finally, it has been also shown that allele-specific Col1a1 and Col1a2 silencing using small interfering RNAs leads to reduction of the mutant collagen in both human and mice cell models (23, 24). These findings should be further replicated and validated before moving into clinic.

Overall, all of these approaches have shown to have possible therapeutic benefit in OI types I-IV. Much less is known about the other forms of OI (types V-XVII) caused by mutations in other genes: *IFITM5* (type V), *SERPINF1* (type VI), *CRTAP* (type VII); *LEPRE1* (type VIII), *PPIB* (type IX), *SERPINH1* (type X), *FKBP10* (type XI), *SP7* (type XII), *BMP1* (type XIII), *TMEM38B* (type XIV), *WNT1* (type XV), and *SPARC* (type XVII).

### Osteopetrosis

Osteopetrosis is a group of rare bone disorders characterized by high bone mass due to defects in osteoclast differentiation and function. The abnormal bone remodeling leads to a variety of skeletal and dental deformities. Moreover, severe forms of osteopetrosis can expand to the bone marrow and the cranial nerve foramina resulting in a plethora of hematological and neurological complications (9). The autosomal dominant form of osteopetrosis (ADO), i.e., Albers-Schönberg disease, is caused primarily by heterozygous mutations in the chloride channel 7 (*CLCN7*) gene (25). On the other hand, there are eight different forms of autosomal recessive osteopetrosis (ARO) [reviewed elsewhere (26)] caused by mutations across several different genes such as *TCIRG1, CAII, OSTM1, TNFSF11, TNFRSF11A* among others.

The clinical diagnosis of osteopetrosis can be easily made using simple X-ray. Nevertheless, it is important to distinguish between the different forms of osteopetrosis using genetic testing as this will determine the choice of disease treatment and prognosis. For instance, patients with CAII deficiency are at high risk of developing nephrocalcinosis and nephrolithiasis (27), whereas patients with mutations in *OSTM1* or *CLCN7* may have severe neurological defects (28). Currently, the only established treatment for the severe forms of osteopetrosis is hematopoietic stem cell transplantation (HSCT). However, the success of the HSCT will depend on the time of diagnosis, i.e., patients undergoing HSCT after the age of 10 months have high prevalence of graft rejection (29). A major issue with HSCT treatment is that it requires HLA-matched donors. For individuals lacking compatible donor, alternative treatment in the future can be gene therapy which has shown promising results in the preclinical trials. For instance, neonatal transplantation of gene-modified HSCs have led to increased bone resorption in oc/oc mice (30, 31). Lentiviral-mediated correction of the genetic defect (*TCIRG1*) have improved osteoclasts activity *in vivo* (32, 33). Last but not least, gene-corrected iPSCs may also be valuable source for testing and developing gene therapy for osteopetrosis (34).

HSCT treatment for *TNFSF11* mutations will be ineffective because in this form of osteopetrosis the main defect does not arise from the haematopoietic lineage (35). Osteopetrosis in TNFSF11-deficient patients requires a different treatment approach. Several pre-clinical studies have shown promising results in the treatment of TNFSF11-dependent osteopetrosis. For instance, the administration of synthetic RANKL to *Tnfsf11*^−/−^ mice led to significant improvement of the bone phenotype (36). In addition, several studies are also testing the use of implants and biomimetic scaffolds as source of synthetic RANKL in order to promote osteoclastogenesis in *Tnfsf11* knock-out mice (37). These methods need to be further validated before entering clinical trials.

Additional novel approaches for the treatment of osteopetrosis include gene silencing using small interfering RNAs (38) and interferon gamma (IFN-y) (39) which have shown to be effective in reducing bone mass. Nevertheless, both approaches need further evaluation before they can be brought to the clinic.

### Sclerosing Bone Disorders

Genetic mutations affecting the *SOST* (chr17q12-21) gene lead to two similar syndromes: sclerosteosis and van Buchem disease, described in detail in previous reviews (10, 40, 41). Sclerosteosis arises from *loss-of-function* mutations within the *SOST* gene; whereas, van Buchem disease from *deletion* of a region (~52-kb) downstream of the *SOST* gene, which is relevant for proper gene expression (42). *SOST* encodes sclerostin, which is a potent inhibitor of the Wnt/β-catenin signaling pathway relevant for osteoblast differentiation and proliferation (43, 44). Moreover, sclerostin incites RANKL secretion and triggers osteoclastogenesis (45). Thus, both diseases are characterized by high bone mass throughout the skeleton. There is no specific therapy for these conditions. The treatment is based on treating symptoms and reducing the severity of complications (40).

The knowledge of the cellular and molecular mechanisms of these rare skeletal disorders have led to new treatment strategies for osteoporosis. The sclerostin monoclonal antibody (Scl-Ab/ Romosozumab), which inhibits the function of sclerostin and enhances bone formation (anabolic compound), is the most recent addition to the osteoporosis set of medications. In the early research stages, Scl-Ab treatment has resulted in dose-dependent increases in cortical and trabecular bone mass and volume at several skeletal sites in monkeys (46) and rats (47). Similar findings were observed during a Romosozumab Phase I clinical trial of 72 postmenopausal women and healthy men (48). In 2014, the phase II clinical trial of Romosozumab showed significant increases in BMD (49), whereas in Phase III (two years later) it was associated with 73% lower risk of future vertebral fracture in women with postmenopausal osteoporosis during 24-months of follow-up (50). Since 2019, Romosozumab is officially approved for clinical use and is promoted as anabolic agent that stimulates bone formation and decreases bone resorption.

It is worth mentioning that when targeting treatments based on gene function (i.e., gain/loss of function mutations) effects can result in opposing outcomes. For instance, *gain-of-function* mutations in the *LRP5* gene result in drastic increases in bone mass (51, 52), while *loss-of-function* mutations cause decreases in bone mass, i.e., osteoporosis-pseudoglioma syndrome (53, 54). Therefore, strong biological knowledge is needed before embarking on clinical trials as provided by pre-clinical cell and animal models, potentially complemented by the rapidly growing number of genome-wide association studies (GWAS) for complex traits. Further, as described below, GWAS of osteoporosis traits have re-identified variants in genes known to harbor mutations responsible for monogenic conditions. This implies that (1) genes identified by GWAS of complex traits serve as prominent candidates to be scrutinized for underlying “unsolved” monogenic conditions; and (2) genes underlying monogenic conditions which are harboring common variants associated with complex traits provide indication of shared biological pathways with enormous translational potential (55).

## What Have We Learned From Complex Skeletal Traits?

### Gene Discovery in Osteoporosis

**Figure 1:**
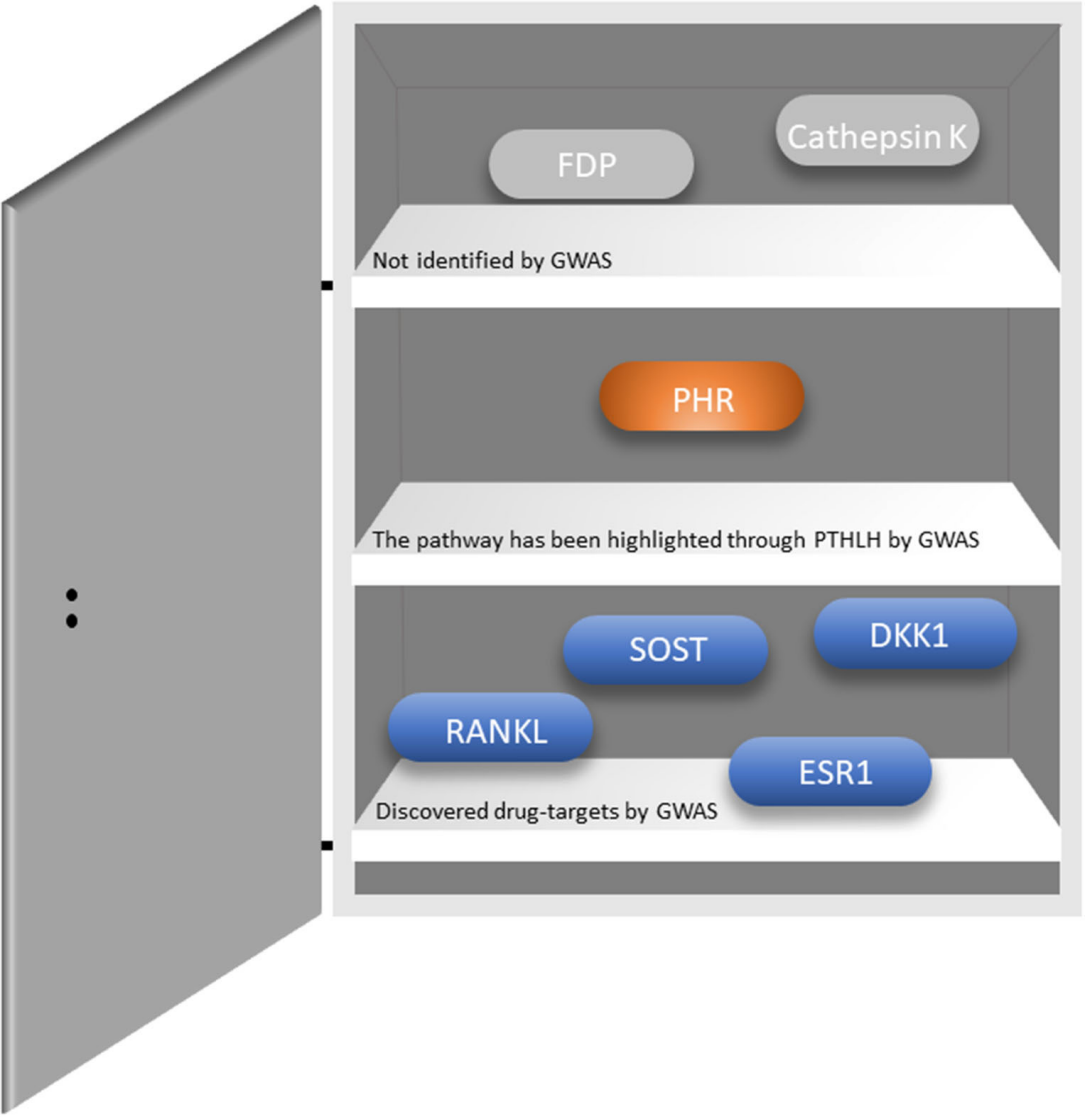
Existing drug targets and whether they have been identified through GWASs. RANKL-Receptor activator of nuclear factor-κB ligand; SOST-sclerostin; DKK1-Dickkopf WNT signaling pathway inhibitor 1; ESR1-Estrogen Receptor 1; PHR-parathyroid hormone receptor; FDP-Farnesyl pyrophosphate. The figure was adapted from Jepsen et al. (63).

Osteoporosis is a common complex skeletal disease with a devastating endpoint i.e., fractures. From a genetic perspective osteoporosis is a systemic multifactorial disease caused by a combination of genetic, environmental, and lifestyle factors. Similarly, fracture risk, comprises a very complex trait involving numerous biological and biomechanical processes that are under convoluted genetic and environmental control (55). GWAS have revolutionized the field of genetics of complex traits and common diseases, where osteoporosis is no exception. There are several reviews describing in detail the findings arising from the Genetic Factors of Osteoporosis (GEFOS) consortium, and the UK Biobank (56–58). Currently, there are close to thousands of variants associated with increased risk of osteoporosis (12, 59–62). These variants map to relevant bone pathways such as Wnt signaling (*LRP5, WNT16, AXIN1, CTNNB1, DKK1, WLS, LRP4, MEF2C, RSPO3, SERP4„ SOST, WNT4, WNT5B, EN1*), OPG-RANK-RANKL (*TNFRSF11B, TNFRSF11A, TNFSF11*) and endochondral ossification (*PTHLH, RUNX2, SPP1, SOX6*), but also novel pathways yet to be explored. Recently, novel genes have been discovered, which are involved in bone metabolism (*SLC8A1, PLC1*, and *ADAMTS5*); osteoblast and osteoclast differentiation and activity (*CSF1, DUSP5, SMAD3, SMAD9*, and *CD44*); and manganese and calcium absorption (*GCKR, DGKD*, and *SLC30A10*), among others (59). Importantly, there is an overlap between the monogenic skeletal conditions and BMD GWAS-identified genes such as *GALNT3* [OMIM: 211900], *COL1A1* [OMIM: 166210 and 259420]*, SHFM1* [OMIM: 601285]*, ESR1* [OMIM: 615363], and *LRP4* [OMIM: 212780]; genes implicated in mesenchymal cell differentiation, skeletal development, and bone remodeling and metabolism (55). Remarkably, GWASs on BMD have identified existing or promising drug targets (Figure 1); reflecting the 267-fold enrichment observed across GWAS for validated drug targets in humans (56). However, such relatively high number of discovered drug targets represents a small fraction of the total number of discovered genes. Hence, many novel drug targets are yet to be identified. Recently, two genes identified by GWAS have been proposed as possible novel drug targets for osteoporosis. Kemp et al. (60) using multiple layers of evidence from bioinformatical, functional and biological knowledge have shown *GPC6* to play a prominent role in bone biology. *Gpc6*^−/−^ mice is characterized by a high bone mass phenotype and increased cortical bone thickness (60). This gene has also been linked to several essential pathways such as fibroblast growth factor, vascular endothelial growth factor, Hedgehog, and bone morphogenetic protein pathways (64). *GPC6* encodes a glypican anchored on the cell membrane in the extracellular matrix and it is relevant for cell signaling. In particular, the heparan sulfate proteoglycans attached to the GPC6 protein regulate skeletal signaling pathways involved in bone formation and mineralization; thus, representing a potential good drug target. In humans, GPC6 is mainly expressed in the liver and bladder tissue. Given the lack of bone and muscle tissues in publicly available databases there is no evidence for its expression in bone tissue. Nevertheless, GPC6 have been reported to be expressed in osteoblasts and osteocytes in mice (60). Yet another potential drug target identified by GWAS is *DAAM2* which regulates canonical Wnt signaling. Further, CRISPR–Cas9-mediated *DAAM2* knockout models in osteoblast cells lines are shown to exert substantial reduction in inducible mineralization (61). To date, only few genes have been linked with bone mineralization, making this gene particularly interesting as drug target. Nevertheless, further investigations are needed to establish whether *GPC6* or *DAAM2* truly represent a suitable drug target for osteoporosis.

Before the GWAS era, genetic research was mainly driven by typically underpowered and ill-defined candidate gene studies. For that purpose, the Genetic Markers for Osteoporosis (GENOMOS) consortium was created in order to study “classical” candidate gene polymorphisms such as *ESR1, COL1A1, VDR, TGFbeta* and *LRP5* in relation to osteoporosis-related outcomes in a well-powered setting of subjects drawn from several study populations around the globe. The main phenotypic outcomes included fracture risk and femoral neck and lumbar spine BMD. The first GENOMOS meta-analysis evaluated three polymorphisms in the *ESR1* gene among 18,917 individuals across eight studies and demonstrated no effects on BMD but a modest effect on fracture risk (19–35% risk reduction for XbaI homozygotes), independent of BMD (65). Next, GENOMOS embarked on studying *COL1A1*, a gene with long standing candidacy to be involved in the etiology of osteoporosis given its established effect on monogenic forms of skeletal fragility, i.e., osteogenesis imperfecta. GENOMOS observed a significantly lower BMD (0.15SD) in TT homozygotes for the *COL1A1* Sp1 polymorphism; (66) as well as a non-significant trend toward a 10% increase in vertebral fracture risk per T-allele. The vitamin D receptor (*VDR*) is yet another long-standing candidate gene of osteoporosis, given the crucial role of vitamin D on bone biology. GENOMOS scrutinized 5 *VDR* polymorphisms (Cdx2, FokI, BsmI, ApaI, TaqI) none of which had evidence for association with either BMD or fracture risk (67). Similarly, none of the 5 tested *TGFbeta* polymorphisms had effect on BMD or fracture risk (68). Last but not least, GENOMOS did demonstrate an effect for *LRP5* coding polymorphisms (Val667Met, Ala1330Val) on BMD as well as on fracture risk, while no effect for the (Ile1062Val) variant in *LRP6* (69). Altogether, the meta-analyses of the GENOMOS has provided high degree of evidence of involvement across what were then regarded as high-risk alleles. Of these, only *ESR1* and *LRP5* have shown to be effectively contributing to explain population variance in risk for osteoporosis. In line with the findings of GENOMOS, none of the classical “osteoporosis” candidate genes, such as *VDR* and *TGFbeta*, have been identified in the GWAS meta-analyses of GEFOS as associated with osteoporosis traits. This is not really surprising and stresses the need for well-powered GWAS and the importance of replication of identified genetic associations.

### The Potential of Polygenic Risk Scores in Osteoporosis

Polygenic risk scores (PRSs), which harness findings from GWAS, defined as a sum of genetic variants associated with a specific trait/disease, have recently evolved with the promise of entering and be implemented in the clinic; among other uses to predict an individual's risk of disease. PRSs have been successfully applied across many complex diseases such as type 2 diabetes (70, 71), coronary artery disease (71, 72), atrial fibrillation (71), inflammatory bowel disease (71), breast cancer and depression; (73) and for some of these conditions characterizing groups of individuals with a risk equivalent to that observed for patients with monogenic mutations (71). Another advantage of the PRS, is that genetic effects are stable across the life-course, therefore holding the potential to predict the onset of disease decades before it occurs. Similarly, genetics can contribute to improved treatment strategies by identifying people who will most benefit from treatment or are at higher risk of adverse effects (74).

In the field of osteoporosis in particular, the use of polygenic risk scores and the Mendelian randomization (MR) approach has brought upon novel insights. In the field of prediction, efforts drawn within the highly-powered setting of the UK Biobank have shown impressive predictive ability. Kim (62) showed that a genetic algorithm using 22,886 SNPs was correlated with heel ultrasound estimated BMD (rho = 0.42) and that combining this genetic algorithm with clinical information could improve this correlation (rho = 0.5). More recently, Forgetta et al. (75) showed that a PRS for heel quantitative ultrasound speed of sound (SOS)—can reassure the low risk of individuals who can safely be excluded from a fracture risk screening program. The “gSOS algorithm” (genetically predicted SOS) employed 21,717 genetic variants that were strongly correlated with standard SOS testing. These findings are important, considering that gSOS proved to have similar predictive power as existing diagnostic tools for osteoporosis i.e., the fracture risk assessment tool (FRAX) with 93.4% and BMD-based FRAX tests with 98.5%. These findings suggest that such genetic tool can help to confidently exclude low risk patients from undergoing osteoporosis testing and its associated health care costs.

Just as in other fields, the field of osteoporosis has witnessed how genetic data arising from the GWAS have brought many successes in linking traits and diseases to provide more robust evidence of causality, i.e., when used in a MR framework (76). Randomized Clinical Trials (RCTs) are the gold standard for testing whether exposure is causal for a specific outcome. However, it is not always easy to conduct a RCT. From this perspective, MR has emerged as a promising approach to address this. As our genotypes are randomized by nature (Mendel's second law of independent assortment), the MR approach has been developed to derive more robust evidence of causal association between risk factors and outcomes. MR uses genetic variants that are fixed at birth as instrumental variables in order to derive un-confounded causal effect estimates for modifiable risk factors. Most importantly, performing an MR analysis prior to an RCT can be helpful to predict adverse effects and/or unexpected outcomes, sometimes helping decide if it is worth launching the RCT or not. The use of the MR approach in the field of osteoporosis has provided remarkable insight when scrutinizing the main clinical risk factors of fracture risk. A plethora of risk factors for osteoporosis identified by observational studies have been tested using MR [reviewed by us and others elsewhere (77, 78)]. Trajanoska et al. (79) showed that BMD is the most important “causally-related” determinant of fracture risk; and that prevention strategies aimed at increasing or maintaining BMD are the most likely to be successful, in contrast to strategies targeting the other traditionally used risk factors. Remarkably, the MR analysis showed how continuous increases in vitamin D levels are not causally related with fracture risk; providing robust evidence showing that indiscriminate vitamin D supplementation in the general population is unlikely to be effective for the prevention of fracture. This work and the follow-up work by Trajanoska et al. (79) and Cerani et al. (80) showed that calcium intake or calcium levels are also not causally related with fracture risk. The MR analysis also showed that such genetically-derived increase in serum calcium is associated with an increased risk of coronary artery disease (81). These findings imply that widespread calcium supplementation in the general population has a questionable risk/benefit ratio.

### Pharmacogenetic Studies in Osteoporosis

From a clinical perspective there are several pharmacological alternatives for osteoporosis such as bisphosphonates, selective estrogen receptor modulators (SERMs), Hormone replacement therapy (HRT), Teriparatide and Denosumab, among others. About 10–15% of osteoporosis patients fail to gain bone mass (>3%) despite receiving anti-osteoporotic therapy (82–85). Treatment non-response can be due to poor adherence, duration and type of treatment but can also represent a biologically-based failure. Stratified or personalized approaches can be quite relevant for complex disease where an individual's drug response will depend on the combination of markers that regulate absorption, availability, activity and metabolism of drug compounds (86). There is little data about the pharmacogenetics of osteoporosis and osteoporotic fractures. In the past, research has mainly focused on few candidate genes, namely the *VDR*, estrogen receptor alpha (ER-α) and *COL1A1* genes, which have been investigated with regard to response to osteoporosis-drugs. Long before GWAS, some drug-gene interaction with bisphosphonates have been postulated. For instance, VDR variants were suggested to modify the effect of alendronate (87, 88) or of etidronate (89); the *COL1A1 Sp1* polymorphism the effect of etidronate; and variants of genes belonging to the *FDPS* (mevalonate pathway) the response to amino-bisphosphonate treatment (90–92). Allelic combinations of *SOST, PTH, FDPS*, and *GGPS1* gene variants may also have a role in the individual response to bisphosphonate treatment (93). From the other clinically-relevant perspective of side effects, several polymorphisms mapping within the cytochrome P450-2C (*CYP2C8)* gene have been associated with increased risk of bisphosphonate-induced osteonecrosis of the jaw in patients with multiple myeloma (94). While illustrating the great potential underlying potential pharmacogenomic investigations, these results need to be interpreted with caution. These studies have been performed in underpowered settings and the tested variants have not been robustly replicated across larger GWAS. Thus, additional evidence from large-scale GWAS aimed at investigating gene x drug interactions are needed, ideally combined with knowledge from cell and animal models to robustly ascertain possible pharmacogenomics effects. Such pharmacogenomic studies represent a clear knowledge gap in the osteoporosis field.

## Current Needs in the Osteoporosis Treatment Framework

Currently we are facing challenges in osteoporosis care. The operative “decision-to-treat” definition of the disease currently relies on the BMD measurement, transformed to a sex-matched young-adult (peak-bone mass acquisition age) reference in the form of T-scores (95). Pragmatically, individuals with a T-score BMD measurement < −2.5 standard deviations (SD) of the BMD of a young adult are classified as having osteoporosis; and due for the initiation of pharmacological treatment and control of risk factors. Individuals with a T-score between −2.5 and −1.0 are classified as having osteopenia and (in absence of clinical risk factors or fractures) pharmacological treatment is not indicated while preventive life style reinforcement is provided. This strategy is extremely suboptimal, as more than 50% of the fractures occur above the osteoporosis threshold (96). Nevertheless, risk assessments tools that incorporate clinical risk factors on fracture risk over and above the risk provided by BMD, are guiding clinicians in identifying individuals at high fracture risk, so that treatments can be targeted. For example, FRAX (University of Sheffield) integrates clinical risk factors and femoral neck BMD to calculate the 10-year probability of suffering hip fracture and any major osteoporotic fracture (clinical spine, forearm, hip, or shoulder fracture) (97). The predictive ability of FRAX using clinical risk factors and BMD is effective in helping to identify gradients of risk and make decisions on treatment indication. Nevertheless, the current clinical framework still falls short in its ability to predict accurately response to treatment and providing tailored approaches to maximize the effectiveness of treatments. As discussed above, addition of genetic information to the prediction models can lead to improved risk stratification.

The osteoporosis field possesses multiple pharmacologic therapeutic options at hand, where at least 46 trials (comprising 138,523 participants) have been performed since 1990, including studies on: Bisphosphonates: alendronate (98–106), clodronate (107, 108), etidronate (109), risedronate (110–113), and since more recently zoledronate (114–119), and ibandronate; (120, 120) HRT: estrogen (121) (+progestin) (122) and since more recently, tibolone; (123) SERMs: raloxifen, (124, 125, 125) lasofoxifene (126), bazedoxifene (127, 128), arzoxifene; (129) Calcitonin; (130) Cathepsin K inhibitors: odanacatib; (131) RANKL inhibitor: denosumab; (132–134) Strontium; PTH and analogs: abaloparatide, (135) teriparatide, (136) PTH (1-34) (137, 138) and PTH (1-84); (139) and an Anti-sclerostin antibody: romozosumab (140).

These sets of compounds can be broadly classified into bone resorption inhibitors (including bisphosphonates, HRT, SERMs, Calcitonin and RANKL inhibitor) and bone formation (anabolic) agents (including Anti-Sclerostin antibody, Strontium and PTH/PTH-analogs though the latter two can also exert anti-resorption effects). Even with the diverse treatment alternatives, there is a so-called “crisis in the treatment of osteoporosis” i.e., despite the existence of effective drugs to prevent fractures, patients, including those assessed as having a latent high fracture risk and who unequivocally need treatment, are not prescribed osteoporosis medications or they refuse to take them given the uncertainty surrounding the prolonged use of anti-resorptive medications and under the fear of ominous rare adverse events (like increased risk of osteonecrosis of the jaw and atypical femur fractures) (141). The current clinical framework lacks crucial information on the biologic and mechanistic pathways underlying the complex nature of fracture risk that are not contemplated in the current T-score based definition of osteoporosis. Altogether, rather than a primary need to embark in the search of “superior” treatments, there is a current need to scrutinize the performance of- and adherence to- current osteoporosis therapies. The best way to do these is by bringing new knowledge resulting in an operative molecular redefinition of osteoporosis that will improve patient care by bringing the field closer to personalized/tailored interventions for the prevention and treatment of the disease.

## Genomic Medicine in Osteoporosis Practice

### Future Use of Polygenic Risk Scores (PRS) in Clinical Practice

**Figure 2:**
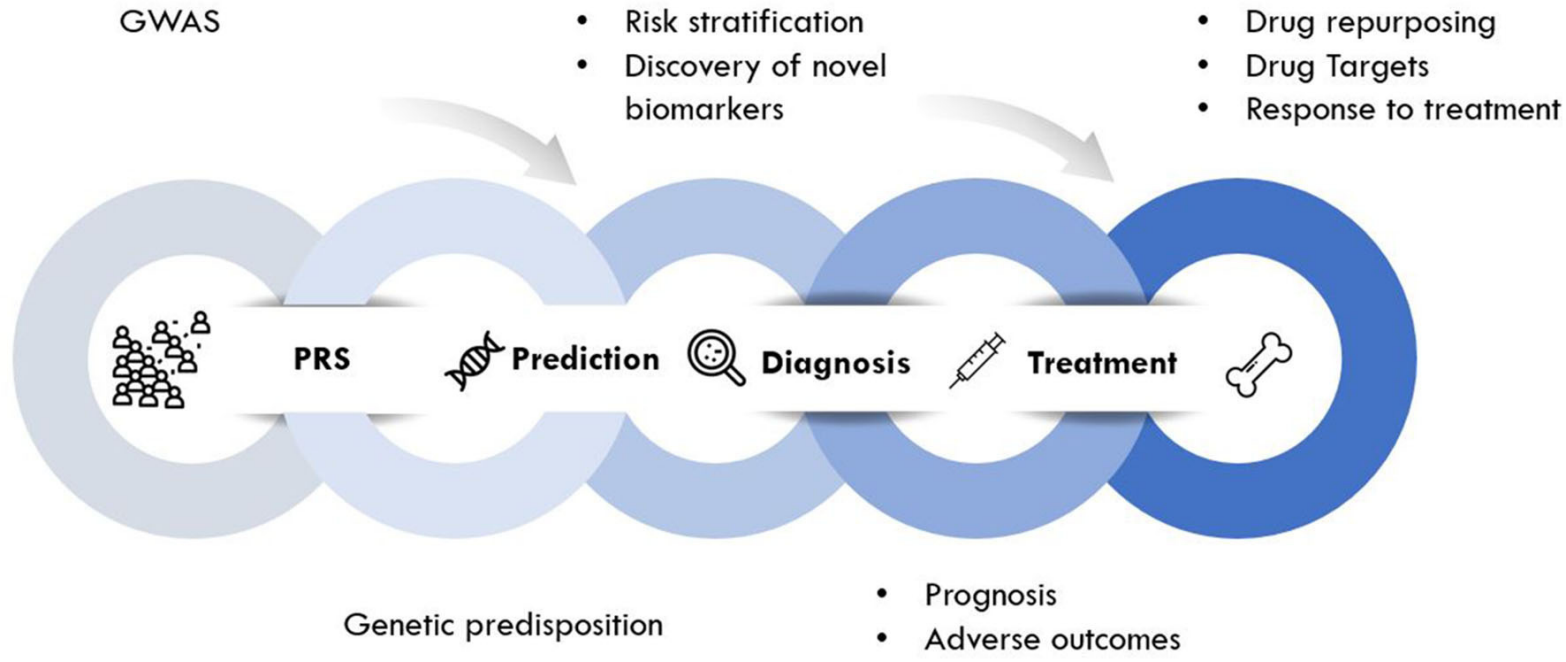
Implementation of genetic information in clinical care.

**Figure 3:**
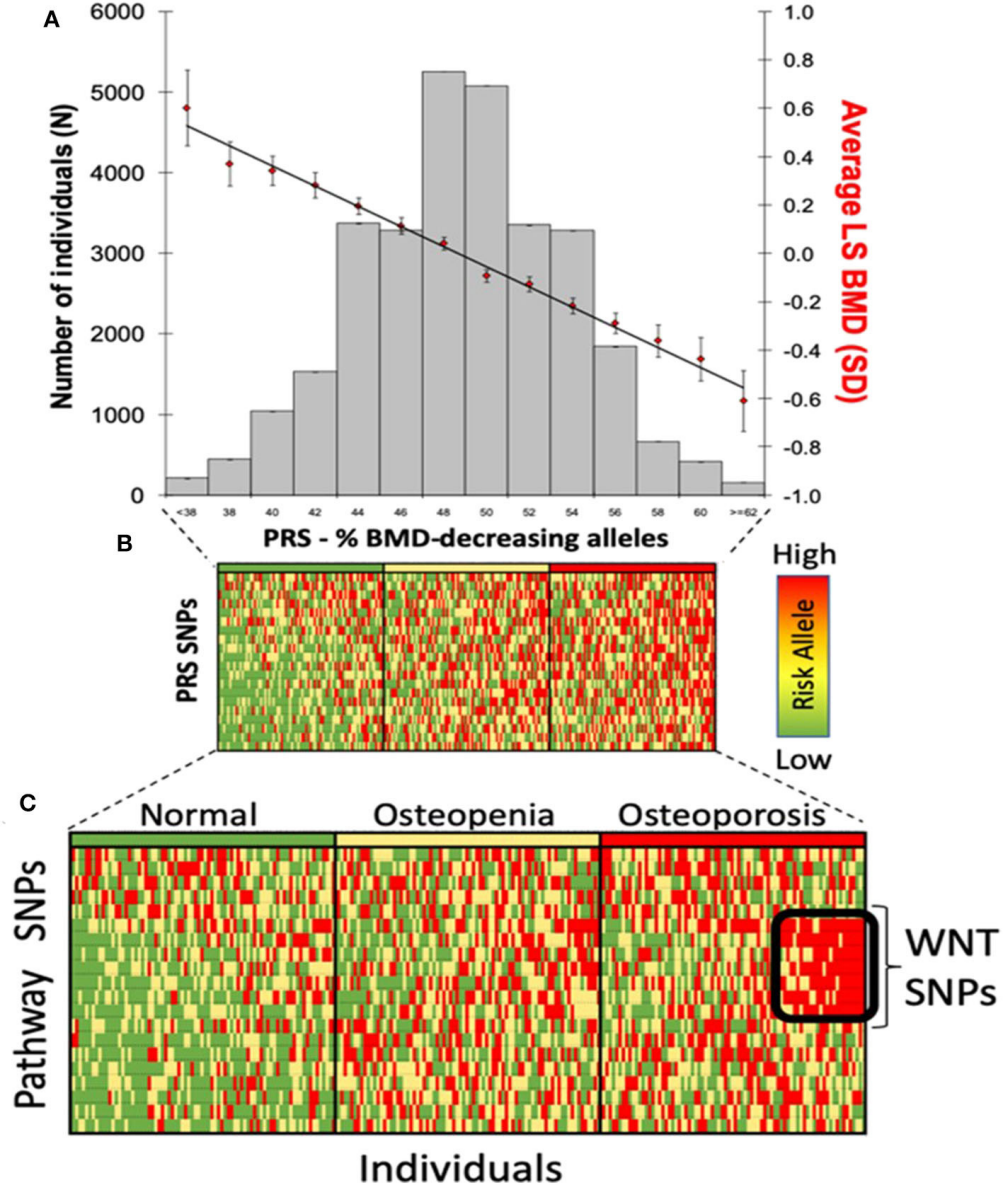
Polygenic risk scores in clinical practice. **(A)** distribution of PRS in the population; **(B)** disease risk stratification according to risk allele distribution; and **(C)** risk stratification using biological pathways.

Genomic approaches are key to the development of personalized medicine as they contribute to: (1) the understanding of disease at a molecular level; (2) the identification of new biomarkers (quantifiable parameters of disease development or disease prognosis); and (3) optimization of therapeutic interventions (drug target discovery, novel indications or response to treatment) (Figure 2). Genomic rather than seeking the identification of a unique treatment beneficial for each individual person, tools of genomic medicine primarily seek sub-dividing patients into groups based on their “molecular make-up,” e.g., using PRS. PRS typically follow a “normal distribution” of risk alleles in the population (Figure 3A), with the majority of individuals tending to have a mean number of risk alleles. Toward both sides of the PRS distribution, a lower fraction of the population consists of individuals with either a *very high* or *very low* number of risk alleles. At more extreme truncates of the distribution, disease classification becomes more robust, as it becomes evident that high and low risk alleles underlie (yet not without error) substantial risk differences among groups (Figure 3B); i.e., more risk genotypes (red) cluster to the right (disease category), while non-risk alleles (green) cluster to the left (normal category). Further, the inclusion of SNP annotation to biologic pathways (Figure 3C) can allow pinpointing clusters of patients that theoretically can respond differently to specific pathways targeted by the osteoporosis treatment. This way, the understanding of disease processes at a molecular level, can guide the search for biomarkers of disease risk and differential response to treatments.

Through this so-called patient “stratification” medical interventions can be more successful when fitted to a specific group on patients instead of using the current approach of “one size fits all.” This is true for all diseases in general, but of particular great potential for several musculoskeletal conditions (86). In the years to come, genomic research will bring novel insights into molecular mechanisms of osteoporosis and may lead to disease definition reevaluation. In addition, the newly acquired knowledge will redefine the disease-disease relationships by highlighting shared molecular mechanisms that may serve as drug targets for two or more diseases (see below “drug repurposing”). This way, systems biology approaches guided by genomic studies can help to improve decision-making in pharmaceutical development in the search for novel biomarkers and therapeutic target.

### Drug Discovery and Repurposing

On average, it takes 12 years and ~1.5 billion dollars for a new drug to complete all three phases of clinical trials before getting marketing approval. Despite the expensive and time intensive efforts, 90% of the drugs fail to pass the clinical trials as a result of poor efficacy and safety issues. Nearly 50% of the failures in Phase II are due to lack of efficacy, whereas 25% are result of high toxicity; (142, 143) even occurring after preclinical models have shown the drugs to be efficient and safe. There are many reasons for the drugs to fail during clinical trials; one of them is that in general drug targets are selected based on their intrinsic “druggability” properties but not their biology. If we don't understand fully the underlying biology of the drug target-disease relationship, unexpected outcomes can occur. Nonetheless, in the past decade positive waves in the drug discovery process have been facilitated by advances in the genomic field supported by a variety of novel computational methods. Nowadays, we have better understanding of the genetic architecture of many diseases and traits and we can utilize this genetic information in drug discovery to pinpoint more efficient and safe drug targets. Nelson et al. have shown that genetic support doubles the drug approval rate (144). These findings were later supported by King et al., which also observed that Phase II and III studies are twice more likely to be successful when genetic data is incorporated in the selection of the drug target (145). For instance, GWAS hits have identified variants in genes encoding pathways targeted by compounds in Phase III or already approved drugs (146, 147). Finan et al. (148) have estimated that around 22% of the 20,300 protein-coding genes annotated in Ensembl version 73 are set to be “druggable.” The authors stratified the druggable gene sets into three groups. Tier 1 consists of approved small molecules, biotherapeutic drugs and clinical-case drug candidate; Tier 2 contains a set of genes encoding targets with known bioactive drug-like small-molecule binding partners as well as those with ≥50% identity (over ≥75% of the sequence) with approved drug target; and Tier 3 is a set of genes encoding coding-secreted or extracellular proteins, i.e., proteins with more distant similarity to approved drug targets, and members of key druggable gene families not already included in tier 1 or 2 (148). Mapping the GWAS results to these protein-coding genes may derive new potential drug–targets and is one of our current active areas of research.

Besides discovering novel drug targets, the drug discovery field has directed attention to novel strategies, e.g., drug repositioning or repurposing, meaning using existing “approved” drugs for new indications. In the past, GWAS have also shown to be a good source for drug repositioning as well. Drug repositioning is possible due to how the GWAS field has ascertained the wide-spread presence of pleiotropy, i.e., when one gene (variant) exerts an effect on more than one disease phenotype or complex trait. It is postulated that around 50% of the discovered GWAS hits have pleiotropic effects (149). Therefore, it is possible for one drug to effectively be used for multiple disease indications. Yet, potential adverse effects need to be explored in detail in the case of antagonistic effects. Nowadays, 35% of the approved drugs targets modulate G protein-coupled receptors (GPCRs); the largest family of membrane receptors (150). Yet, only 16% of the ~800 GPCRs are being currently targeted by existing drugs. Hence, the number of GPCR-targeted drugs, and even types of drugs is expected to dramatically increase in the following years (150).

Across the literature there is lots of evidence for repurposed drugs in other fields. For instance, duloxetine is a selective serotonin and norepinephrine reuptake inhibitor antidepressants (SSNRI) used initially for management of major depressive disorder (MDD). Over the years, new indications for the drug have been proposed, being repurposed for the treatment of fibromyalgia (151), and musculoskeletal and diabetic neuropathic pain (152, 153). Next, denosumab, one of the modern anti-resorptive medications to treat osteoporosis, has been used as adjuvant therapy of giant-cell tumor of bone (GCTB) (154). This tumor consists of cluster of neoplastic mononuclear cells and osteoclast like giant cells which express RANK (155). Denosumab inhibits RANKL, thus, supressing bone turnover by reducing osteoclast derived bone resorption while also eliminating giant cells. Nowadays, there are also ongoing efforts examining benefits of denosumab therapy on other types of cancer. Such efforts seek pinpointing possible mechanisms that prevent bone metastasis and increase bone metastasis free survival. This hypothesis is based on the knowledge that RANKL is connected with a variety of signaling pathways implicated in immunity and cancer. RANKL and it receptors are essential for lymphoid tissue formation, lymphocyte differentiation, dendritic cell survival and T-cell activation (156, 157); all relevant components for proper functioning of the immune system. Recently it has been shown that concomitant therapy of denosumab and immune checkpoint inhibitors (ICI) may also have beneficial effect on cancer survival and progression (158, 159). Therefore, denosumab might have a tremendous clinical impact. In addition, there is ongoing research on the potential anti-cancer mechanisms of bisphosphonates as some (160) but not all (161) clinical trials on breast cancer have reported evidence for anti-tumor effects. This anti-neoplastic effects are plausible, considering that bisphosphonates may alter cellular signaling and responsiveness by modification in the isopentenyl diphosphate metabolism, untimely leading to cell death (162, 163). A meta-analysis of 61 clinical trials has also shown bisphosphonates use to be associated with reduced cardiovascular and all-cause mortality (164). Nevertheless, more research is needed before repurposing approaches become common practice, but there is no doubt about the great underlying potential to enrich the therapeutic landscapes of musculoskeletal disease.

Last but not least, other approaches using “-omics” technologies, such as proteomics and metabolomics can provide additional information about the efficacy of new drug targets, elucidate part of the underlying biology, and most importantly, support strategies to anticipate adverse outcomes (165). In addition, many biological molecules interact with each other and cluster based on particular functions or so-called pathways. There are more than 150 different pathway databases, such as Biocarta, KEGG, and Reactome. These resources can help shed additional light on drug-disease mechanisms, decrease the number of false positives in the drug target identification step, prioritize validation, and aid selecting the best target; altogether helping improve the drug discovery success rate (166). As such, there are a variety of computational approaches to detect novel drug-disease relations supported by multitude of databases [reviewed elsewhere (167, 168)].

### Drug Target Validation Using Mendelian Randomization

MR analysis can be also performed to investigate potential risk factors or prognostic factors, and to evaluate drug targets (169). With the fast growing pace of the genomic field much better genetic instruments have become available, allowing the expansion of MR studies for the identification and validation of potential drug targets and their adverse effects (170). The genetic variants used as instrumental variants have been implicated as encoding drug targets (170). Nowadays, there are several online platforms which provide detailed information about the underlying biology of the gene of interest, such as the Online Catalog of Human Genes and Genetic Disorders (OMIM). Combining GWAS and OMIM can be a useful way to derive genetic instruments and to test the drug directionality for variety of heritable diseases with available data (171, 172). Furthermore, novel methods have been developed to utilize other –*omics* data in order to detect causal drug targets. The recent focus has been made on proteins, which are the target of most drugs, i.e., using proteomics information to leverage drug identification. Schmidt et al. (173) have developed a novel MR framework for drug validation so called “cis-MR” directed at increasing the precision and robustness of the MR approach. Traditionally, MR uses genetic variants as instrumental variables that are associated with the outcome, independent from other genetic variants in the locus or located elsewhere throughout the genome. In contrast, the cis-MR approach is more stringent, only employing genetic variants located in or in the vicinity of a protein coding genes (173). Recently, work by Zheng et al., have highlighted the important role of *cis* and *trans* protein quantitative trait loci (pQTLs) MR analysis, which coupled with evidence for colocalization produces robust evidence of causal protein-phenotype associations as well (174). Next to the aforementioned approaches, phenome-wide MR (MR-PheWAS) can be performed to detect any adverse associations with other clinically relevant outcomes. MR-PheWAS allows testing for a casual association between a selected exposure and a range of phenome-wide disease outcomes; from where we can replicate or discover new relationships between traits or disease outcomes.

A perfect example of a successful MR-drug discovery is the validation of proprotein convertase subtilisin–kexin type 9 *(PCSK9*). *Loss-* and *gain-of-function* genetic variation in this gene region have been associated with low-density lipoprotein cholesterol (LDL-C) an important risk factor for coronary artery disease. Monoclonal antibodies that inhibit *PCSK9* have been proposed as a novel drug target to reduce LDL-C and decrease the risk of cardiovascular events; findings that have been robustly confirmed by RCT (175) and other MR studies (176). Additionally, using the MR approach, variations in *PCSK9* have been also linked to adverse outcomes such as increased risk of type 2 diabetes (177). These findings demand careful consideration and follow-up of patients using PCSK9 inhibitors to treat hypercholesterolemia, who may be at risk of developing diabetes. In the skeletal field there are other examples of the MR success. Interestingly, observational studies showed in the past that low-doses of cholesterol-lowering statin drugs could be associated with higher BMD and decreased risk of fracture (178, 179). Recent MR studies found genetically lower LDL to be associated with higher BMD levels as well (180, 181). In addition, variants mapping to the statins gene-drug target (*HMGCP*) were associated with increase in BMD (180). The effect of *HMGCP* on bone were established to be partly acting through lowering LDL-C. This indicates that other biologic pathways targeted for other conditions, may affect bone and might have a potential therapeutic implication that needs to be examined in the future. It is important to note that MR cannot test for dose dependent associations. Thus, sometimes it is relevant to couple the evidence from MR with the information coming from well-conducted observational studies. Further, it is important to note that MR studies can inform decision making to launch (or not) RCT about interventions. MR studies can provide evidence to RCTs about aspects of efficacy; unexpected favorable outcomes (with repurposing potential); adverse effects of the evaluated targets; and importantly, the risk of RCT failure. Given the large costs of RCTs, assessing the evidence from MR studies should be considered as part of the planning of any RCT trial. Altogether, while these MR approaches are pending to be widely applied in the field of osteoporosis, MR findings pointing to causal relations between risk factor and outcome, coupled with strong biological understanding of the drug-target disease association, will allow robust validation of drug targets.

## Current Progress in Gene Editing for Monogenic and Complex Bone Diseases

Gene editing in clinical practice has major implications in understanding, treating, and preventing deleterious genetic diseases. There are several tools that allow researchers to modify a specific DNA region such as zinc-finer nucleases (ZFNs) (182), transcription activator-like effector nucleases (TALENs) (183), and Clustered Regularly Interspaced Short Palindromic Repeats (CRISPR)/Cas9 (184) [reviewed elsewhere (185, 186)], among others. Compared to the other editing methods, CRISPR/Cas9 has been positioned as faster, cheaper, more precise, and more efficient in selecting and binding to the target DNA sequence. Therefore, the use of the CRISPR/Cas9 technique has become widely spread in the past few years.

Skeletal genetic defects have been typically studied in two animal models, i.e., mouse [reviewed by Maynard et al. (187)] and zebrafish [reviewed by Bergen et al. (188)]. Expectedly, CRISPR/Cas9 gene editing in these mouse and zebrafish models of human skeletal disease has swiftly evolved as comprehensively reviewed, by Wu et al. (189). To date, CRISPR/Cas9 models have been done for several skeletal disorders such as OI (190), meningocele syndrome (191) and campomelic dysplasia (192). OI type V is a rare autosomal dominant disease characterized by increased bone fragility, low BMD and increased susceptibility to bone fracture followed by hyperplastic callus formation. OI type V is caused by heterozygous mutation in the *IFITM5* gene which adds 5 amino acid residues (Met-Ala-Leu-Glu-Pro denoted MALEP) on the N-terminus of the BRIL protein (encoded by *IFITM5*) (193). Rauch et al. (190), generated for the first time, a MALEP-BRIL knock-in mice using CRISPR-cas9. The MALEP-BRIL hetero*zygous* mice presented severe skeletal deformities such as short and bent long bones which lack the primary ossification center. Although the current model was genetically identical to human OI type V, it showed additional clinical manifestations. CRISPR/Cas9 animal models have also provided novel insights in the pathology of osteoporosis. In a small scale study (1,625 Han Chinese) variants mapping to *ATP6V1H* were associated with spine BMD (194). The same study, produced a *Atp6v1h*^+/−^ knockout mice using CRISPR/Cas9 which presented with decreased bone remodeling accompanied by impaired bone formation and increased bone resorption (194). Similar findings were observed in atp6v1h^+/−^ zebrafish generated by CRISPR/Cas9 as well (195). Last but not least, there have been several advances in osteogenic gene therapy that has shown promising results in bone remodeling and fracture healing [reviewed elsewhere (196)].

## Conclusions

Genomic studies are and will continue to be an inexhaustible source of information to better understand the genetic underpinnings of both monogenic and complex bone disorders. In the long run, genetic discoveries will have vast clinical implications, paving the road to precision medicine. Genomic medicine will support clinical-decision making and will dramatically improve disease screening, diagnosis, prognosis and treatment.

## Author Contributions

All authors listed have made a substantial, direct and intellectual contribution to the work, and approved it for publication.

## Conflict of Interest

The authors declare that the research was conducted in the absence of any commercial or financial relationships that could be construed as a potential conflict of interest.
